# A case of pediatric livedoid vasculopathy: Diagnostic and management challenges

**DOI:** 10.1016/j.jdcr.2026.06.057

**Published:** 2026-07-02

**Authors:** Jamie Warner Kahli, Leeandra Cleaver, Daniel Valladares, Renee Modica, Kiran Motaparthi

**Affiliations:** a University of Florida (UF) College of Medicine, Gainesville, Florida; b UF Department of Pediatric Rheumatology, Gainesville, Florida; c UF Department of Pediatrics, Gainesville, Florida; d UF Department of Rheumatology, Gainesville, Florida; e UF Department of Dermatology, Gainesville, Florida

**Keywords:** direct oral anticoagulants (DOACs), factor Xa inhibitor, immunoglobulins, intravenous (IVIg), livedoid vasculopathy, pediatric dermatology, rivaroxaban, thrombotic vasculopathy

A 12-year-old White female with no significant medical or family history developed a painful rash involving the dorsolateral feet and ankles starting at 8 years of age. The rash evolved from bruise-like areas to ulcers and then resolved with scarring and hyperpigmentation. New lesions appeared each spring and summer, improved in fall, and remitted in winter. During flares, pain prevented participation in recreational activities, limited ambulation, and resulted in visits to the emergency department. Previous treatments, including topical and oral antibiotics as well as mometasone ointment, were ineffective.

At presentation, physical exam demonstrated retiform purpura, scarring, and ulcers in a reticulate configuration on the dorsolateral feet (Fig 1). The clinical diagnostic impression of livedoid vasculopathy (LV) was confirmed by skin biopsy of new purpura, which demonstrated a thrombotic vasculopathy affecting small vessels with hyalinization of vessel walls (Fig 2). Antiphospholipid antibodies (beta-2 glycoprotein, anticardiolipin, phosphatidylserine), dRVVT, ATIII activity, plasminogen activity, plasminogen activator inhibitor 1 activity, prothrombin mutation, prothrombin time–international normalized ratio, activated partial thromboplastin time, Factor V Leiden, protein C/S, homocysteine, lipoprotein A, rheumatoid factor, antinuclear antibody, cryoglobulin, cryofibrinogen, antineutrophil cytoplasmic antibody screen, myeloperoxidase/Pr-3 antibodies, immunoglobulin levels (immunoglobulin G, immunoglobulin M, and immunoglobulin A), C3, and C4 were all normal.

**Fig 1:**
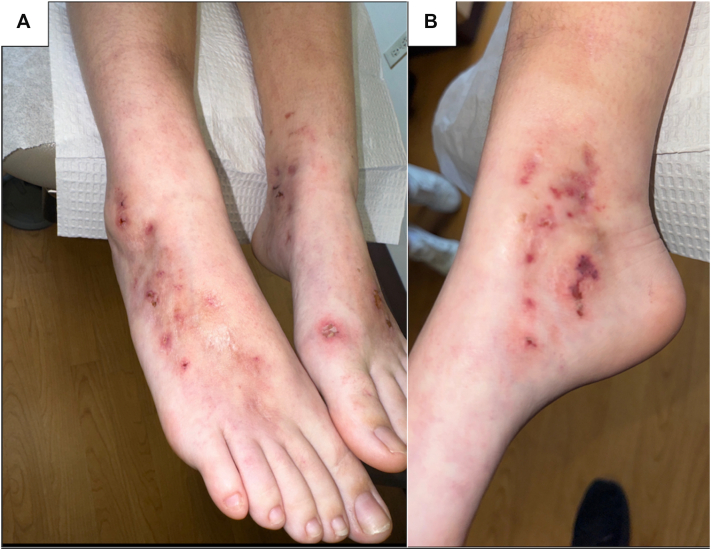
**A,** Presentation to the ED with purpura and ulcers of bilateral feet and ankles. **B,** Punch biopsy was taken of the newest purpura, located below the right medial malleolus.

**Fig 2:**
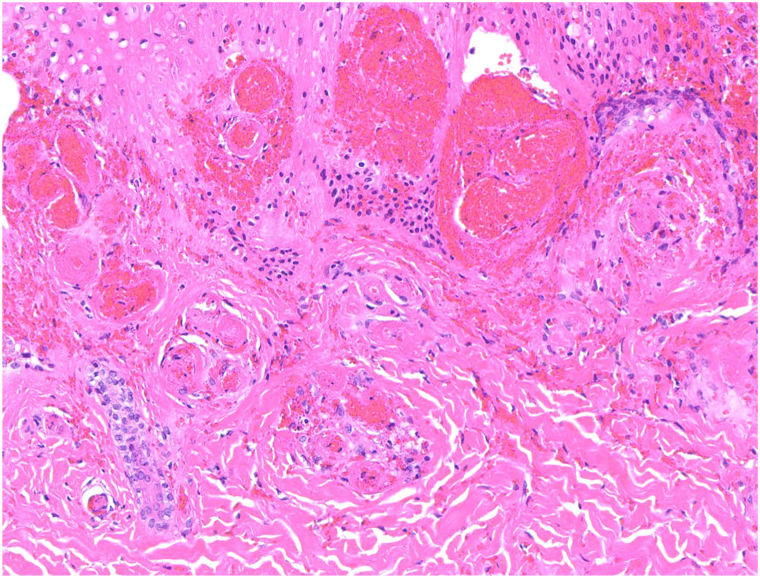
Nonvasculitic occlusive vasculopathy affecting small vessels of the superficial dermis and characterized by hyaline appearance and necrosis of vessel walls, fibrin thrombi, and absent inflammation (H&E, 200× magnification).

The patient was started on aspirin 162 mg once daily and intravenous immunoglobulin (IVIg), receiving 1 cycle per month for 3 months. The first cycle was dosed at 2 g/kg, but for patient tolerance the dosing of the next 2 cycles was reduced to 1 g/kg. By the third cycle, the purpura and ulcers had resolved completely, with only scar and dyspigmentation remaining (Fig 3). At that point, the plan was to reserve IVIg for flare-ups.

**Fig 3:**
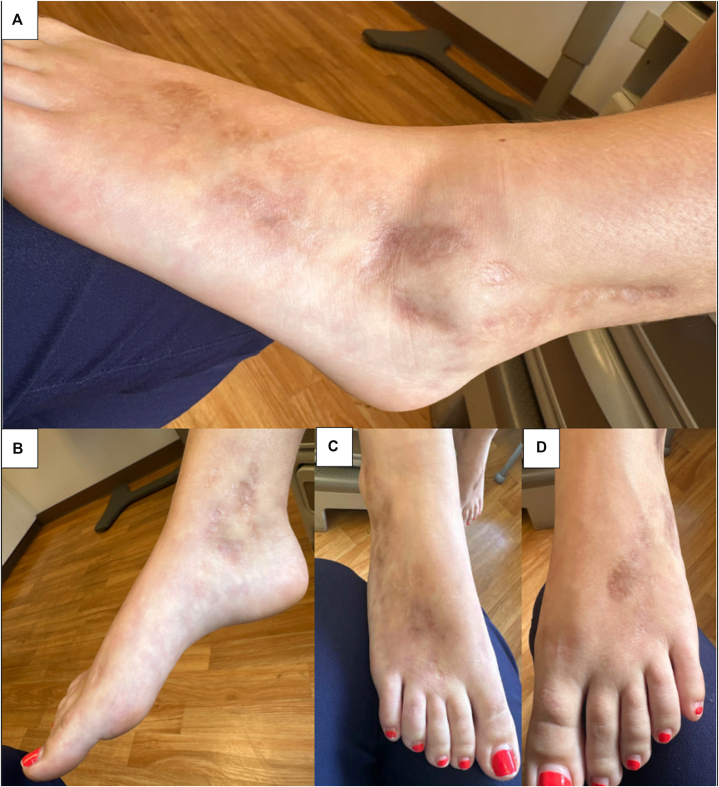
Continued improvement with only mild hyperpigmentation and scar **(A)** 1 month, **(B)** 2 months, and **(C,D)** 6 months out from her fifth and final cycle of IVIg while on rivaroxaban.

Twelve weeks after her third cycle of IVIg, the patient experienced a flare which was treated with a fourth cycle (1 g/kg) of IVIg with plan for 1 g/kg IVIg in 2 months, and rivaroxaban 10 mg twice daily was added to the prior regimen of aspirin 162 mg once daily. Two months after her flare, she had no new lesions and prior lesions had healed. She received her final dose of 1 g/kg IVIg. Aspirin was discontinued as there were no active lesions. She remains on rivaroxaban 20 mg once daily and she has not required rescue treatment with IVIg. She continues to be free from disease 9 months out from her last flare and has not had any adverse events on rivaroxaban.

The only treatments for LV supported by clinical trials are IVIg and rivaroxaban.^1^^,^^2^ Specifically, IVIg has shown efficacy for adult LV in 2 separate clinical trials.^1^^,^^2^ Rivaroxaban has been validated in adults through both trial and observational data,^1^^,^^3^ and a retrospective study confirmed its role in relapse prevention.^4^ There have, however, been no clinical trials on LV treatment in children. Therefore, we conducted a PRISMA-compliant scoping review to see if treatment of pediatric LV with both IVIg and anticoagulation existed in other types of research studies. Using PubMed (search completed May 22, 2026 with terms: “livedoid vasculopathy or “Livedoid Vasculopathy”[Mesh]) AND (“Platelet Aggregation Inhibitors”[Mesh] or “Anticoagulants”[Mesh] or antiplatelet or anticoagulant or anticoagulation”) with a filter to include only human subjects, and 269 articles resulted.

Only 1 study was found that mentioned IVIg. A 17-year-old patient presented with symptoms since 12 years old with flares in the summer—similar to our patient-- and prior treatments of pentoxifylline, danazol, and prostaglandin E-1.^5^ They underwent a trial of IVIg 2 g/kg divided over 3-5 days monthly for 3 cycles but experienced recurrence after 17 months and were then administered 1 cycle of IVIg 2 g/kg, after which lesions remained stable for 10 months.^5^

Further literature review revealed 4 articles containing outcome data specific to patients under 18 years old with LV treated with anticoagulants or antiplatelet therapy other than acetylsalicylic acid, but without the mention of IVIg.^6^ One case report described a 13-year-old boy with LV treated with intravenous heparin (dosage and duration not specified) and rivaroxaban 10 mg daily which resulted in substantial pain relief within 1 week and marked improvement in the ulcers after 2 months of rivaroxaban therapy.^7^ Another case report included a 14-year-old girl with LV and elevated lipoprotein(a) who was treated with enoxaparin 1 mg/kg daily and experienced pain improvement within 7 days and resolution of ulceration by 4 months. Enoxaparin was then discontinued, with recurrence of pain and ulceration; therapy was restarted, and the lesions resolved again.^8^ Three female patients, aged 15-24 years, from a case series of patients with longstanding, recalcitrant LV were treated with rivaroxaban 10 mg daily. For all 3 patients, pain improved within 1 week and vasculopathic findings improved significantly by week 4. All patients continued therapy, with no recurrence reported during follow-up of up to 1 year.^3^ Lastly, a 17-year-old boy with recalcitrant LV was reported whose pain improved within days of starting edoxaban 15 mg daily, with complete ulcer healing by 8 weeks.^9^

Based on our scoping review, this report appears to be the first documented case of pediatric LV treated with both IVIg and rivaroxaban. This case demonstrates promise for the addition of rivaroxaban in pediatric LV cases, especially those recalcitrant to IVIg alone. Given the cost-effectiveness and convenience of rivaroxaban, this treatment should be considered in children with LV.

## Conflicts of interest

None disclosed.
